# Aurora A Phosphorylation of YY1 during Mitosis Inactivates its DNA Binding Activity

**DOI:** 10.1038/s41598-017-10935-5

**Published:** 2017-08-30

**Authors:** Karen E. Alexander, Raed Rizkallah

**Affiliations:** Department of Biomedical Sciences, Florida State University, Tallahassee, Florida United States of America

## Abstract

Successful execution of mitotic cell division requires the tight synchronisation of numerous biochemical pathways. The underlying mechanisms that govern chromosome segregation have been thoroughly investigated. However, the mechanisms that regulate transcription factors in coordination with mitotic progression remain poorly understood. In this report, we identify the transcription factor YY1 as a novel mitotic substrate for the Aurora A kinase, a key regulator of critical mitotic events, like centrosome maturation and spindle formation. Using *in vitro* kinase assays, we show that Aurora A directly phosphorylates YY1 at serine 365 in the DNA-binding domain. Using a new phospho-specific antibody, we show that YY1 phosphorylation at serine 365 occurs during mitosis, and that this phosphorylation is significantly reduced upon inhibition of Aurora A. Furthermore, we show, using electrophoretic mobility shift and chromatin immunoprecipitation assays, that phosphorylation of YY1 at this site abolishes its DNA binding activity *in vitro* and *in vivo*. In conformity with this loss of binding activity, phosphorylated YY1 also loses its transctivation ability as demonstrated by a luciferase reporter assay. These results uncover a novel mechanism that implicates Aurora A in the mitotic inactivation of transcription factors.

## Introduction

The central goal of mitotic cell division is the faithful distribution of the replicated genetic material from a mother cell to two daughter cells. This process requires major morphological changes at the cellular level, like chromatin condensation, breakdown of the nuclear envelope, and formation of the mitotic spindle^1^. The tight coordination and synchronisation of a tremendous number of biochemical reactions is needed to achieve the successful execution of these sequential steps. Deregulation of even a few of these pathways could result in mitotic catastrophe and cell death or, alternatively, propagation of subtle genomic instabilities that can lead to lethal diseases, like cancer^2–4^.

Active transcription, which usually takes place in interphase and involves the formation of large multiprotein complexes on exposed DNA, is not compatible with these events. It has long been known that there is a transient and global suppression of transcription during mitosis^5–7^. For several decades, it was presumed that mitotically-condensed chromatin is simply inaccessible to transcription factors. Consequently, this aspect of mitotic regulation remained scarcely investigated, particularly as compared to the mechanisms of chromosome segregation. However, more recently, we have witnessed a rekindled interest in the mitosis-specific regulation of transcription factors. This came from discoveries that these DNA-binding proteins do not just passively dissociate from mitotic chromosomes. Rather, many transcription factors have been shown to be actively regulated during mitosis^8–11^. Certain regulatory mechanisms completely inactivate the DNA binding activity of some of these proteins, allowing mitosis to be a window for the reestablishment of gene expression programs^12^. On the other hand, some pools of transcription factors remain associated with chromatin all through mitosis to maintain some transcriptional memory at certain loci, a mechanism known as mitotic bookmarking^13^. Moreover, there is now evidence that some transcription factors, that localise to the cytoplasm during mitosis, are post-translationally modified to gain important new functions. These modified proteins can then directly contribute to the integrity of critical steps of mitosis, like centrosome maturation and spindle formation^14^. Therefore, the mitosis-specific regulation of transcription factors is an important aspect of cell division. Further investigation of these mechanisms is needed for a complete understanding of this basic biological process.

The majority of mitotic events are governed by serine/threonine phosphorylation signaling pathways. Massive waves of phosphorylation and de-phosphorylation events orchestrate the entry into and exit from mitosis, respectively^15^. The matured activity of the cyclin-dependent kinase Cdk1 is the key trigger for the initiation of mitosis. However, several other enzymes like Polo-like kinase 1 and the Aurora kinases are also critical for normal cell division^16^. Aurora A in particular has been shown to play important roles in proper centrosome maturation, spindle formation, and chromosome attachment and segregation^17, 18^. Aurora A has gained significant interest because of its upregulation in many cancers, and has been proposed as a promising therapeutic target^19^. Hence, there have been significant efforts to elucidate all of its functional activities and to identify its substrates and *in vivo* target sites^20–22^. Recently, Aurora A was also implicated in the mitosis-specific inactivation of transcription factors through the phosphorylation of the RUNX proteins^14^. Here, we identify a novel involvement of Aurora A in this aspect of mitotic regulation through the phosphorylation of the transcription factor Yin Yang 1 (YY1).

YY1 is an essential and ubiquitously expressed multifunctional protein needed for the cell’s most basic biological pathways^23–25^. Complete ablation of YY1 results in peri-implantation lethality in mice, whereas its partial ablation results in severe developmental defects^26^. As a transcription factor, YY1 has been shown to bind hundreds of DNA sites and to regulate a very large number of target genes with a wide range of functionalities, including cell growth, proliferation, differentiation, metabolism, DNA repair, and even apoptosis^23, 24^. Interestingly, knockdown of YY1 causes the emergence of di- and multi-nucleated cells, indicative of cytokinesis failure^27^. These observations propose a potential role for YY1 in the regulation of cell division. However, it is not currently clear whether YY1 is directly involved in the mitotic process or indirectly through the G2/M transcriptional regulation of mitotic proteins^27^. We have previously shown that, as cells enter mitosis, YY1 loses its DNA binding activity and that the majority of the YY1 protein dissociates from mitotic chromosomes. YY1 regains its DNA binding activity and rapidly re-associates with chromatin at telophase^28^. In conformity with this re-association, YY1 has been shown to be needed for the reactivation of a set of genes at the M/G1 stage, which marks the re-entry into interphase^29^. Therefore, YY1 is transcriptionally active and can bind target DNA sequences at the entry and exit of mitosis, but not during mitosis. Here, we identify serine 365 residue in the DNA-binding domain of YY1 as a mitotic phosphorylation site that can completely inactivate its DNA binding activity. We provide evidence that this site on YY1 is an *in vitro* and *in vivo* novel substrate for Aurora A.

## Results

### Aurora A phosphorylates YY1 *in vitro* on serine residue 365 in the DNA-binding domain

**Figure 1:**
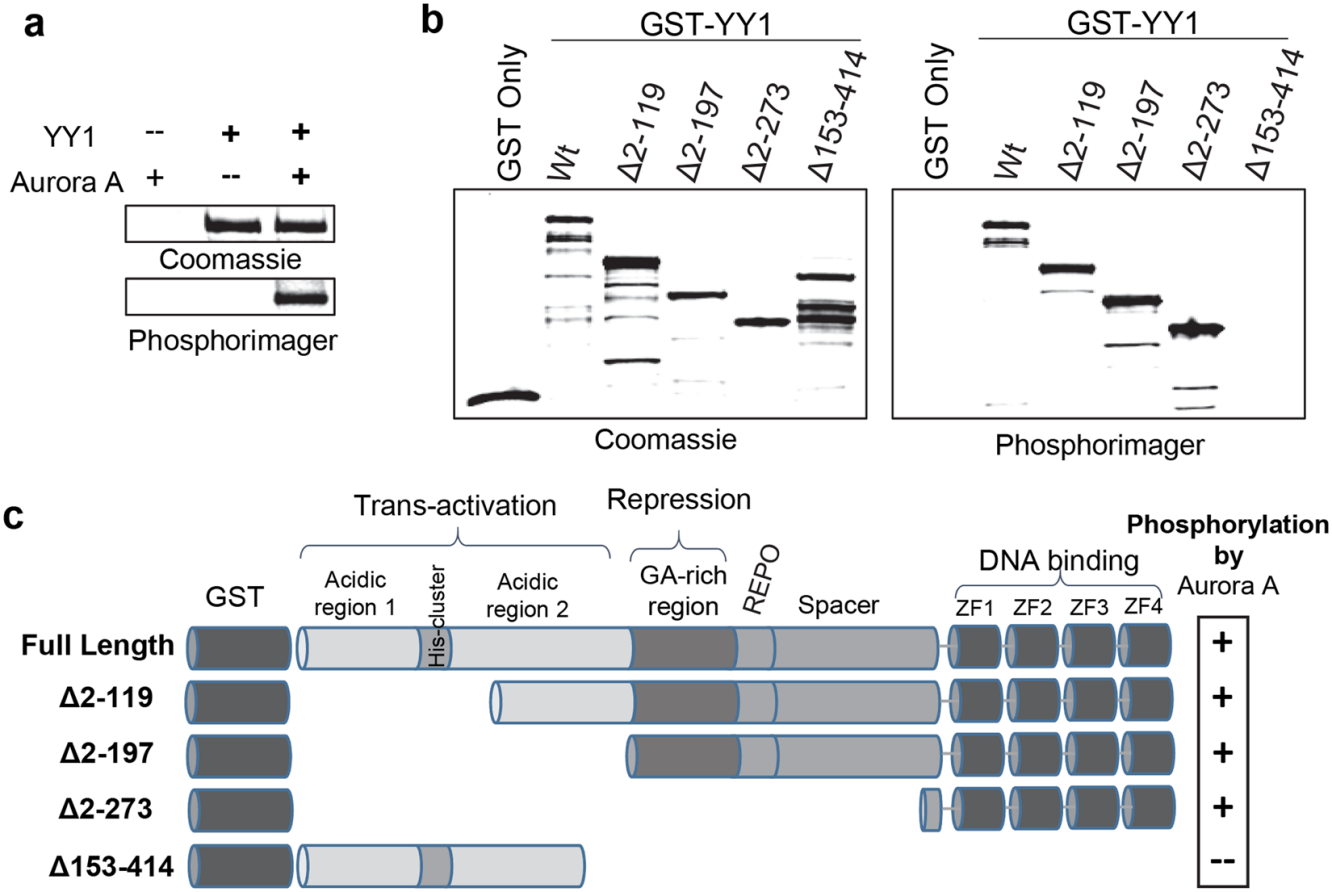
Aurora A phosphorylates YY1 *in vitro* in its DNA-binding domain. **(a)** Radioactive *in vitro* kinase assay using bacterially-expressed and purified YY1 with active Aurora A kinase. After completion, the reactions were separated by SDS-PAGE electrophoresis. The gel was stained with Coomassie blue, dried, and then exposed to a phosphor-imager screen to detect the radioactive phospho-labelling on YY1. **(b)** Radioactive kinase assay as in (**a**) but using bacterially expressed and purified GST or GST-YY1 full-length or deletion mutants as substrates for Aurora A phosphorylation. **(c)** Schematic illustration of the YY1 full length and deletion mutants used in (**b**). The presence or absence of Aurora A phosphorylation is indicated to the right. The structural and functional domains of YY1 are labelled on the top^33, 34^.

Our research has been focused on the investigation of pathways that regulate YY1, particularly through phosphorylation. To identify kinases that can directly phosphorylate YY1, we performed *in vitro* kinase assay screens, using bacterially-expressed YY1 and dozens of commercially available active kinases. These analyses led us to the identification of several kinases that regulate YY1, in a cell cycle-dependent and independent manner, like Polo-like kinase 1 (Plk1) and Casein Kinase II (CKII), respectively^30, 31^. We also found that YY1 is a good *in vitro* substrate for the Aurora kinases. In a previous report, we showed that Aurora B phosphorylates YY1 in the transcriptional repression domain, at late S and G2/M phases^32^. This phosphorylation is likely important for YY1’s activation of genes needed for the G2/M transition. Our screens also showed that YY1 is a good substrate for Aurora A. This is illustrated in Fig. 1a that shows a radioactive *in vitro* kinase assay with purified active Aurora A kinase and bacterially-expressed (non-tagged) YY1.

To investigate whether this phosphorylation mechanism occurs in cells and understand its functional effects, we first needed to identify the Aurora A phosphorylation site on YY1. For this, we purified a panel of bacterially-expressed, GST-tagged, YY1 deletion mutants and tested them as substrates for Aurora A. As shown in Fig. 1b, Aurora A was able to phosphorylate full-length GST-YY1, but not GST. Aurora A also efficiently phosphorylated all of the YY1 N-terminal deletion mutants (Δ 2-119, Δ 2-197, Δ 2-273), but not the YY1 mutant that lacks the C-terminal domain (Δ153–414). A schematic illustration of the YY1 structural and functional domains is shown in Fig. 1c. The N-terminal part of YY1 encompasses its transcriptional activation and repression domains, whereas the C-terminal part contains 4 C2H2 zinc finger domains needed for its DNA binding activity^33, 34^.

**Figure 2:**
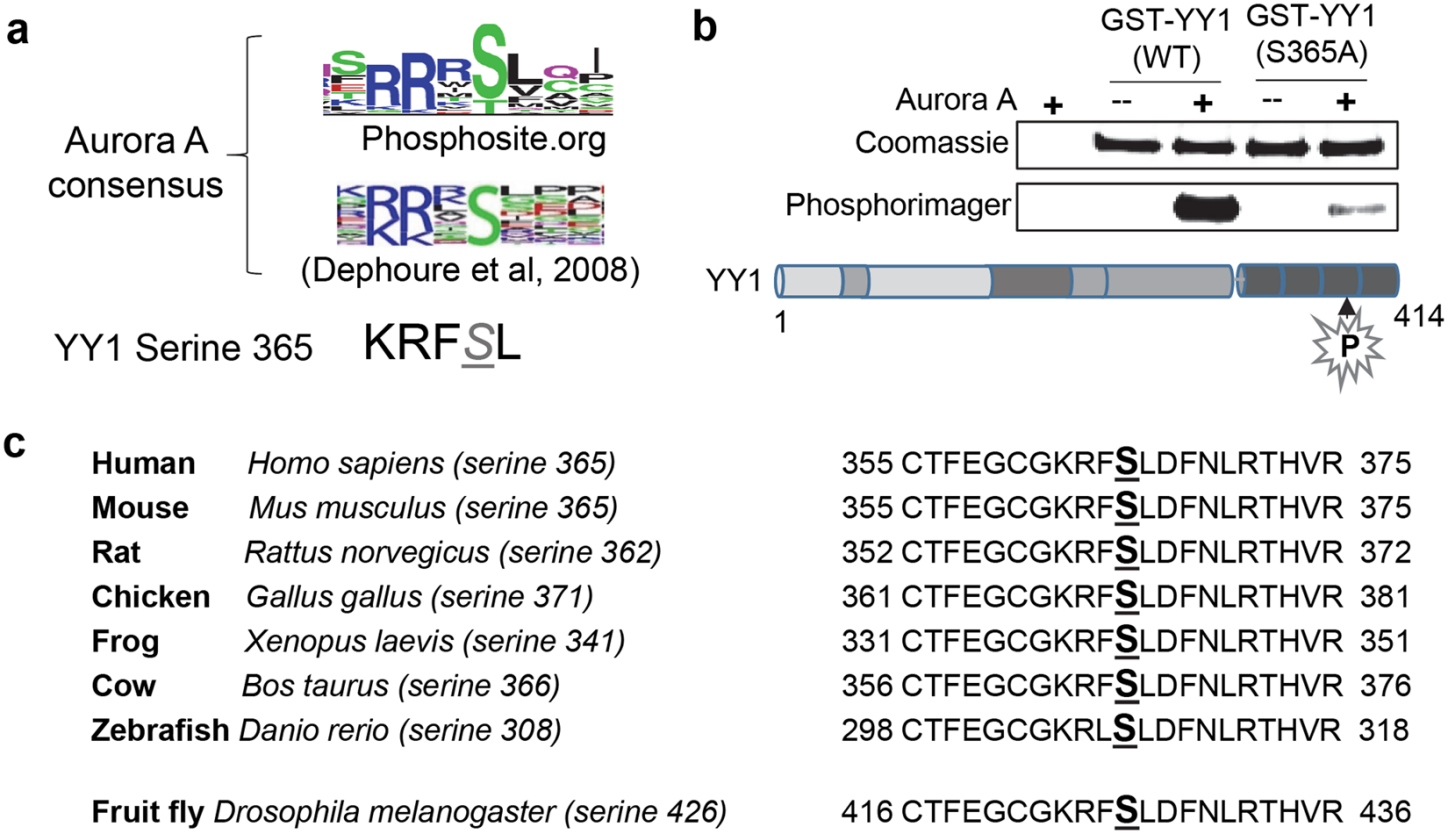
Aurora A phosphorylates the conserved YY1 residue serine 365. **(a)** Comparison of Aurora A consensus phosphorylation motif to the surrounding sequence of serine 365 residue of YY1. The Aurora A motif represented here was generated from empirically demonstrated Aurora A substrates using the phosphosite.org website and as deduced by Dephoure *et al*.^35^. **(b)** Radioactive *in vitro* kinase assay using bacterially-expressed and purified GST-YY1 WT or S365A mutant, with active Aurora A kinase. After completion, the reactions were separated by SDS-PAGE electrophoresis. The gels were stained with Coomassie blue, dried, and then exposed to a phosphorimager screen to detect the radioactive phospho-labelling on YY1. The location of the serine 365 residue on the YY1 protein is illustrated in the diagram. **(c)** Amino acid sequence alignment showing the conservation of the serine 365 residue in YY1, and its surrounding sequence, across various species, as indicated.

To identify the exact residue phosphorylated by Aurora A, we performed a sequence analysis of YY1’s DNA-binding domain in comparison to Aurora A’s previously discovered phosphorylation sites. First, we used the phosphosite.org online database to generate an Aurora A consensus phosphorylation motif based on a large number of empirically-identified Aurora A *in vivo* phosphorylation sites. Second, we examined the Aurora A consensus phosphorylation sequence reported in multiple publications^21, 35, 36^. As shown in Fig. 2a, the Aurora A motif very closely resembles the sequence surrounding serine 365 in the C-terminal domain of YY1. This sequence contains the critical arginine at the −2 position as well as a positively charged lysine at −3 position. In addition, leucine at the +1 position is common in Aurora A consensus sites. Therefore, we postulated that S365 is the most likely target of Aurora A phosphorylation. To test this, we mutated serine 365 to the non-phosphorylatable alanine residue (S365A). Bacterially expressed GST-YY1 WT or S365A mutant was used as substrates in a radioactive *in vitro* kinase assay with Aurora A (Fig. 2b). Coomassie Blue staining shows equal amounts of WT and S365A YY1. The phosphor-imager analysis shows that S365A mutation abolished most of YY1’s phosphorylation. This indicates that serine 365 is the main Aurora A phosphorylation site. An analysis of YY1 amino acid sequence across different species shows that serine 365 and its surrounding amino acids are highly conserved, even in the YY1 *Drosophila* homologue (Pho) (Fig. 2c).

### Serine 365 of YY1 is an *in vivo* phospho-site and occurs in mitosis

Next, we wanted to study the *in vivo* occurrence of serine 365 phosphorylation. First, we searched several published phosphorylation data sets generated from high-throughput mass spectrometry analyses. We found that several of these studies have mapped a phospho-modification on peptides matching YY1 serine 365^21, 37–39^. Therefore, the *in vivo* phosphorylation of this site has been previously confirmed experimentally.

**Figure 3:**
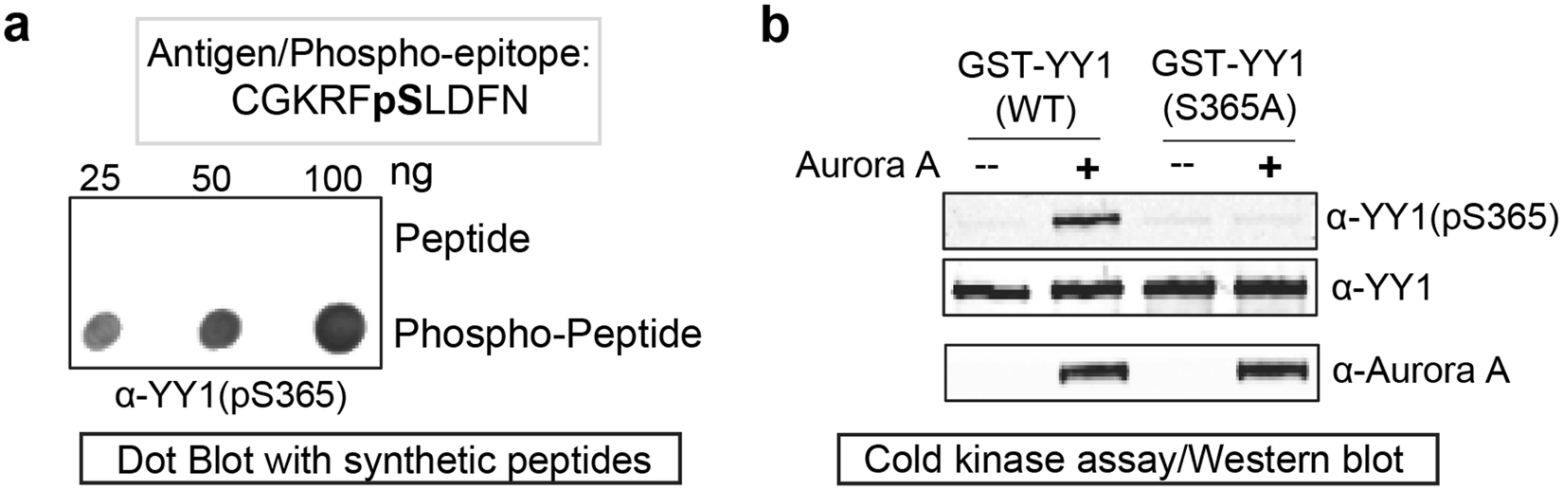
Testing of the anti-YY1 (pS365) phosphospecific antibody. **(a)** Top: amino acid sequence of the phospho-peptide used to generate the novel α-YY1(pS365) antibody. Bottom: The synthetic peptide or phospho-peptide were dot blotted onto a nitrocellulose membrane and probed with the α-YY1(pS365) antibody. **(b)** Cold (non-radioactive) *in vitro* kinase assay using bacterially-expressed and purified GST-YY1 WT or S365A mutant with active Aurora A kinase. After completion, the reactions were separated by SDS-PAGE electrophoresis, transferred onto a nitrocellulose membrane and probed with α-YY1(pS365), α-YY1, and α-Aurora A antibodies, as indicated.

To enable the detailed analysis of serine 365 phosphorylation on YY1 from human protein extracts, we raised a rabbit polyclonal phospho-specific antibody against the epitope CGKRFpSLDFN, comprised of amino acids 360-369 of YY1 with synthetically phosphorylated S365 (α-YY1pS365). To test the phospho-specificity of this new antibody, we first dotted synthetic peptides, phosphorylated and non-phosphorylated, on a nitrocellulose membrane. As shown in Fig. 3a, α-YY1pS365 antibody efficiently recognised the phosphorylated, but not the non-phosphorylated, peptide (Fig. 3a). Then, we wanted to test if the α-YY1pS365 antibody can recognise the Aurora A phosphorylation on YY1. For this, bacterially expressed GST-YY1 WT or S365A mutant were incubated with and without active Aurora A in a cold *in vitro* kinase assay. Reactions were then analysed on a western blot. Figure 3b shows that equal amounts of total YY1 WT and S365A was detected using a commercially available monoclonal antibody against YY1. However, the α-YY1pS365 antibody only recognised YY1 that is phosphorylated by Aurora A. Importantly, α-YY1pS365 could not detect GST-YY1 S365A, even after its incubation with Aurora A. This result clearly shows both the phospho-specificity of the new antibody and its specificity to serine residue 365.

**Figure 4:**
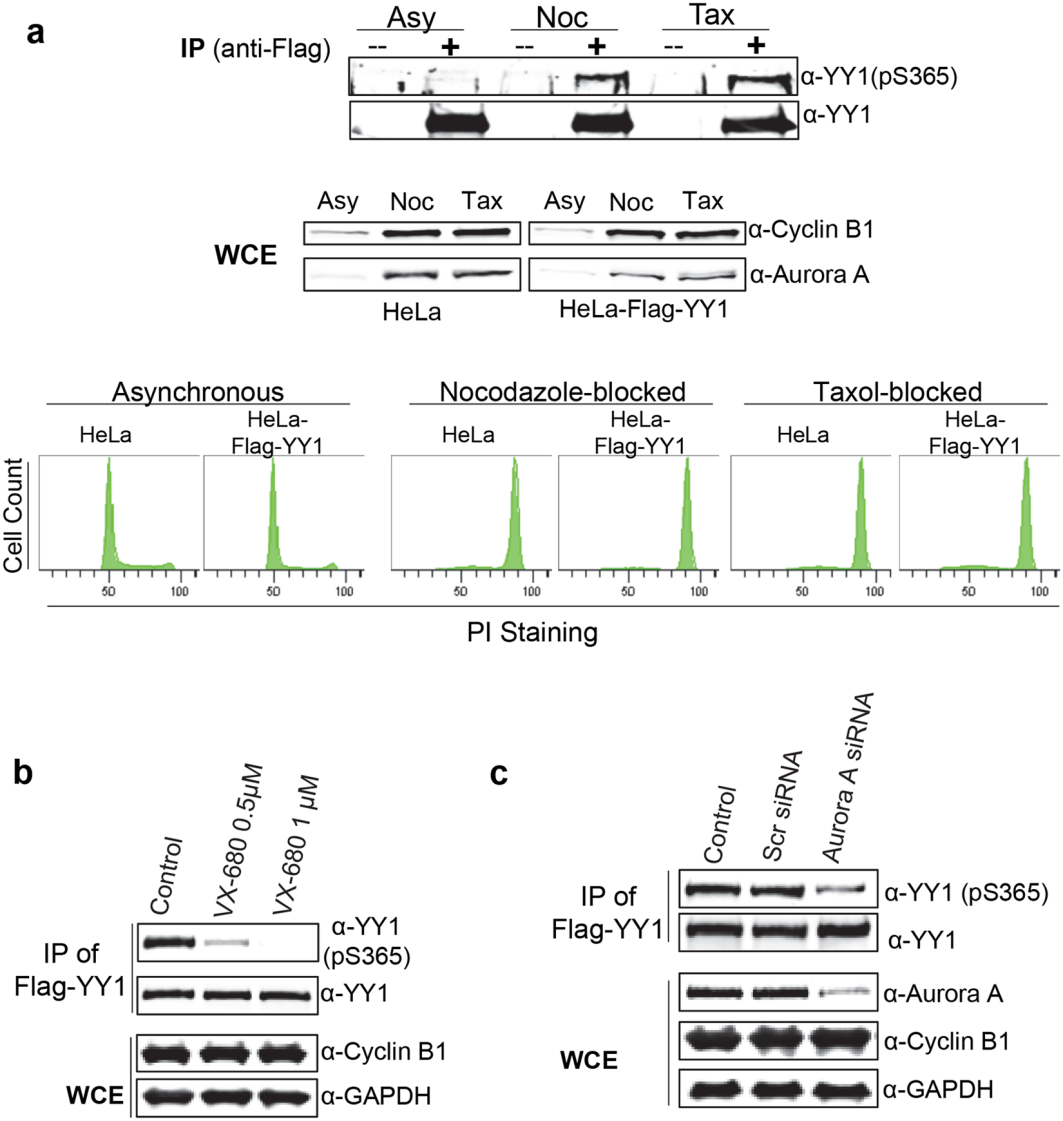
YY1 serine 365 residue is a mitotic Aurora A phosphorylation site *in vivo*. **(a)** Whole cell extracts were prepared from HeLa cells and HeLa cells stably expressing Flag-YY1, growing asynchronously or blocked in mitosis by nocodazole or taxol. Flag-YY1 was immunoprecipitated from whole cell extracts and analysed by western blotting with α-YY1(pS365) and α-YY1 antibodies. Samples from the whole cell extracts (inputs) were also analysed by western blotting with α-Cyclin B and α-Aurora A antibodies. A fraction of cells from each sample was fixed and stained with propidium iodide. Cell cycle distribution was examined using flow cytometric analysis to control for cell synchronization. (**b)** HeLa-Flag-YY1 cells were synchronised in mitosis by nocodazole, then treated with 0.5 or 1 µM VX-680 (or equal volume of DMSO/control) for 15 minutes to inhibit Aurora activity. Whole cell extracts were prepared. Flag-YY1 was immunoprecipitated and analysed by western blotting with α-YY1(pS365) and α-YY1 antibodies. Whole cell extracts were also analysed for Cyclin B levels. GAPDH levels served as a loading control. **(c)** HeLa-Flag-YY1 cells were transfected with scrambled siRNA or Aurora A siRNA (control cells received only transfection reagent). 24 hours post-transfection, cells were synchronised in mitosis by nocodazole for 18 hours and whole cell extracts were prepared. Flag-YY1 was immunoprecipitated and analysed by western blotting with α-YY1(pS365) and α-YY1 antibodies. Whole cell extracts were also analysed for Cyclin B levels. GAPDH levels served as a loading control.

After demonstrating the specificity of anti-pS365 antibody for phosphorylated YY1 *in vitro*, we examined whether phosphorylation of YY1 can be detected *in vivo*. However, we could not efficiently detect this phosphorylation on YY1 immunoprecipitated from protein extracts prepared from an asynchronous cell population. Since Aurora A activity peaks in mitosis^40^, we reasoned that phosphorylation of YY1 at S365 is also likely to be mitotic. Therefore, we compared the phosphorylation of YY1 at S365 in asynchronous and mitotic cells. We used HeLa cells stably expressing Flag-YY1 (HeLa-Flag-YY1) which were described earlier^28^. Protein extracts were prepared from cells either growing asynchronously or arrested at pro-metaphase of mitosis with nocodazole or Taxol treatment. Flag-YY1 was immunoprecipitated from whole cell extracts using mouse monoclonal anti-flag antibody coupled to agarose resin (M2 resin), and analysed by western blotting. Blots were probed with α-YY1pS365 antibody and a general α-YY1 antibody. As expected, equal amounts of total YY1 were detected in extracts from asynchronous or mitotic cells, since YY1 levels remain constant throughout the cell cycle^28, 41, 42^. However, YY1-S365 phosphorylation was only detected in mitotic extracts (Fig. 4a). Samples from the whole protein extracts were tested on a western blot and showed the high levels of Cyclin B and Aurora A in the extracts prepared from mitotic cells compared to asynchronous cells. The synchrony of these cells was further confirmed by analysing a fraction of them using propidium iodide staining and flow cytometric analysis (Fig. 4a, bottom).

### Serine 365 is an Aurora A phosphorylation site *in vivo*

The detection of phospho-serine 365 only in mitotic cells correlates well with the peak of Aurora A activity^40^. However, to have direct evidence that this site is an Aurora A substrate, we analysed this phosphorylation after inhibition of Aurora A. For this, nocodazole-arrested HeLa-Flag-YY1 cells were treated with VX-680, an Aurora kinase inhibitor, for 15 minutes prior to harvest. Flag-YY1 was immunoprecipitated from whole cell extracts with anti-flag antibody and analysed on a western blot. Blots were probed with α-YY1pS365 antibody and a general α-YY1 antibody. Treatment with VX-680 at both 0.5 and 1.0 μM (Fig. 4b) very efficiently inhibited the S365 phosphorylation while total YY1 levels remained constant. We confirmed that the cells did not exit mitosis by examining the Cyclin B levels, which would decrease in case of mitotic slippage.

To conclusively confirm that Aurora A is the *in vivo* kinase for S365 phosphorylation we transfected HeLa-Flag YY1 cells with Aurora A siRNA 24 hours prior to addition of nocodazole. Flag-YY1 was immunoprecipitated from WCE with anti-flag antibody and analysed by western blotting with α-YY1pS365 antibody and a general α-YY1 antibody. Mitotic cells transfected with siRNA targeting Aurora A had significantly reduced levels of S365 phosphorylation (Fig. 4c) as compared to the robust phosphorylation of YY1-S365 detected in mitotic cells in two control samples which included no treatment, or scrambled siRNA. As a control, Cyclin B levels were examined and found to be the same in all the three samples, suggesting that the reduction of YY1pS365 phosphorylation cannot be attributed to an altered cell cycle synchronisation.

Taken together, these *in vivo* Aurora inhibition analyses, the temporal correlation, and the direct *in vitro* phosphorylation assays provide solid support that serine S365 of YY1 is a *bona fide* Aurora A substrate.

### S365 phosphorylation abolishes YY1’s DNA binding activity

**Figure 5:**
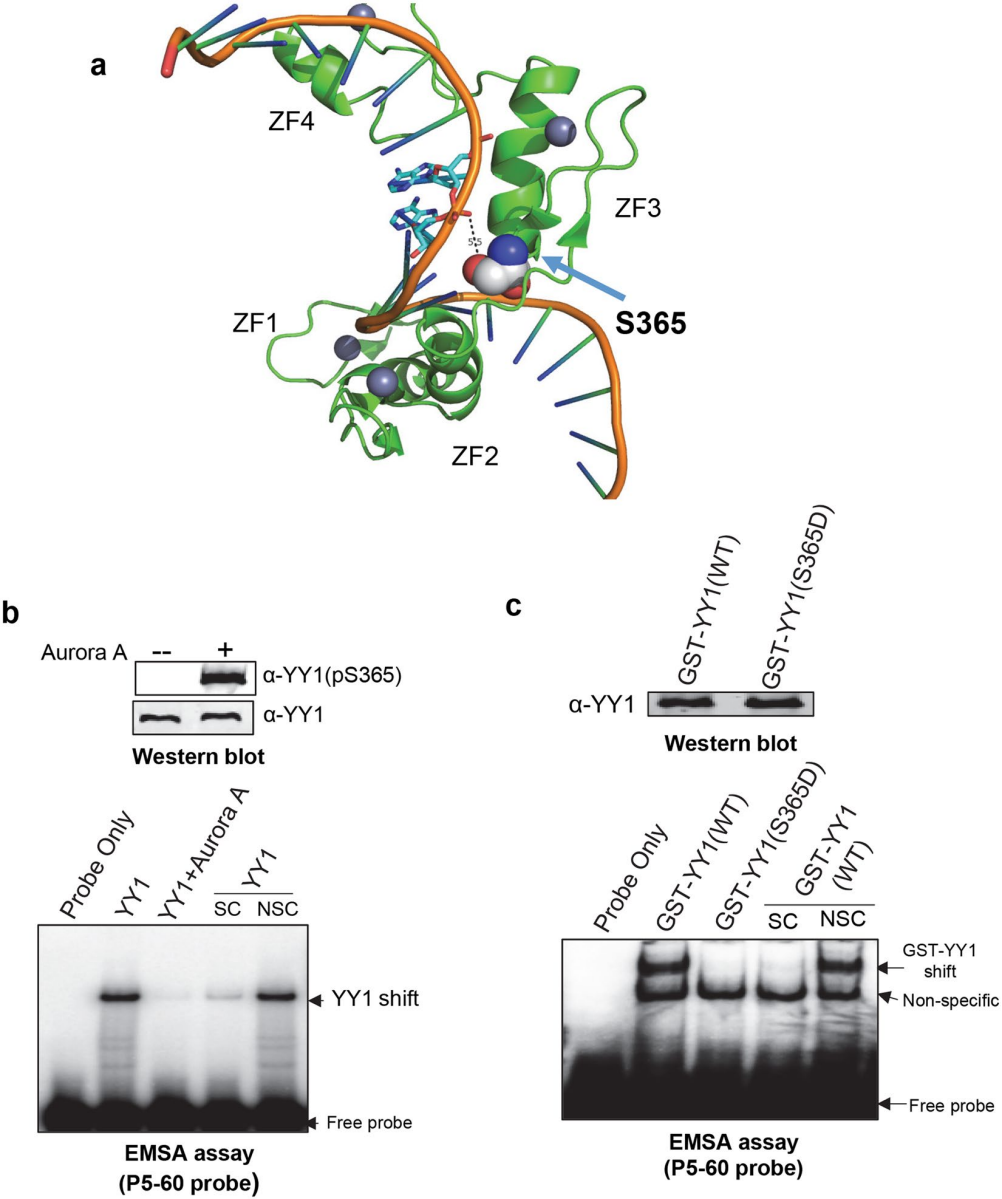
Aurora A phosphorylation of YY1 at serine 365 abolishes its DNA binding activity. **(a)** Illustration of the co-crystal structure of YY1 wrapped around its consensus DNA-binding site in the adeno-associated virus (AAV) initiator element^43^. Image was rendered using Pymol with template PDB 1UBD. The four YY1 zinc finger (ZF) domains and the position of serine residue 365 within the DNA-binding domain are indicated. Serine residue 365 is shown in space-filling mode. The S365 oxygen (red sphere) is approximately 5.5 Å from the phosphodiester backbone. In the structure, a water molecule (not shown) forms a hydrogen bond between the S365 side chain oxygen and the phosphodiester oxygen. The complementary DNA strand is omitted for clarity. **(b)** Bacterially-expressed and purified YY1 was used as a substrate in *in vitro* kinase reactions, with or without active Aurora A. A fraction of the reactions were separated by SDS-PAGE electrophoresis, transferred onto a nitrocellulose membrane and probed with α-YY1 (pS365) and α-YY1 antibodies (upper panel). The remaining fractions of the kinase reactions were tested in an EMSA assay for YY1 binding activity (lower panel). The radioactively labeled probe used here (P5-60) is a double-stranded DNA oligonucleotide containing the YY1 consensus-binding site in the promoter of the adeno-associated virus (AAV). **(c)** GST-YY1 WT or S365D were expressed in bacterial BL21 cells. Bacterial lysates were separated by SDS-PAGE electrophoresis, transferred onto a nitrocellulose membrane and probed with α-YY1 antibody (upper panel). Corresponding amounts of the bacterial lysates were tested in an EMSA assay for YY1 binding activity (lower panel), similarly to (**b**). SC: specific-competition with unlabeled oligonucleotide; NSC: non-specific competition with unlabeled mutated oligonucleotide.

YY1 contains 4 zinc finger motifs that are all needed for efficient DNA binding activity^33^. The serine 365 residue is located in the third zinc finger domain. This specific residue has been found to make an important contact with the DNA backbone as revealed in the co-crystal structure of YY1 bound to the adeno-associated virus (AAV) P5 initiator (Fig. 5a)^43^. Therefore, it is very likely that phosphorylation of YY1 on this site could impact its DNA binding activity. To test this, first we performed an *in vitro* kinase assay with bacterially expressed and purified, non-tagged, YY1 and Aurora A. The phosphorylation of YY1 at S365 was confirmed by analysing the kinase reactions using western blotting (Fig. 5b). We then tested the YY1 DNA binding activity in these kinase reactions in electrophoretic mobility shift assays (EMSA). For this analysis, we used three radioactively labelled DNA probes containing three different variants of the YY1 consensus binding site: AAVP5 + 1, AAVP5-60, and H3.2α^44, 45^. As shown in Fig. 5b (and Supplementary Figure 1a), phosphorylation of YY1 by Aurora A abolished its DNA binding activity. To exclude the possibility that this loss of YY1 binding activity was due to an Aurora A inhibitory interaction with YY1 (rather than phosphorylation), we generated a phospho-mimetic YY1 mutant. Substitution of the serine residue with an aspartic acid residue (S365D) generates a negative charge at that location, and mimics constitutive YY1 phosphorylation. We then tested bacterial lysates expressing GST-YY1 WT or S365D mutant in EMSA assays. As shown in Fig. 5c (and Supplementary Figure 1b), the aspartic acid substitution completely abolished the ability of YY1 to bind DNA on all three different variants of the YY1 consensus binding sites. The equal protein levels of GST-YY1 WT and S365D mutant in the bacterial lysates was confirmed by western blotting (Fig. 5c).

**Figure 6:**
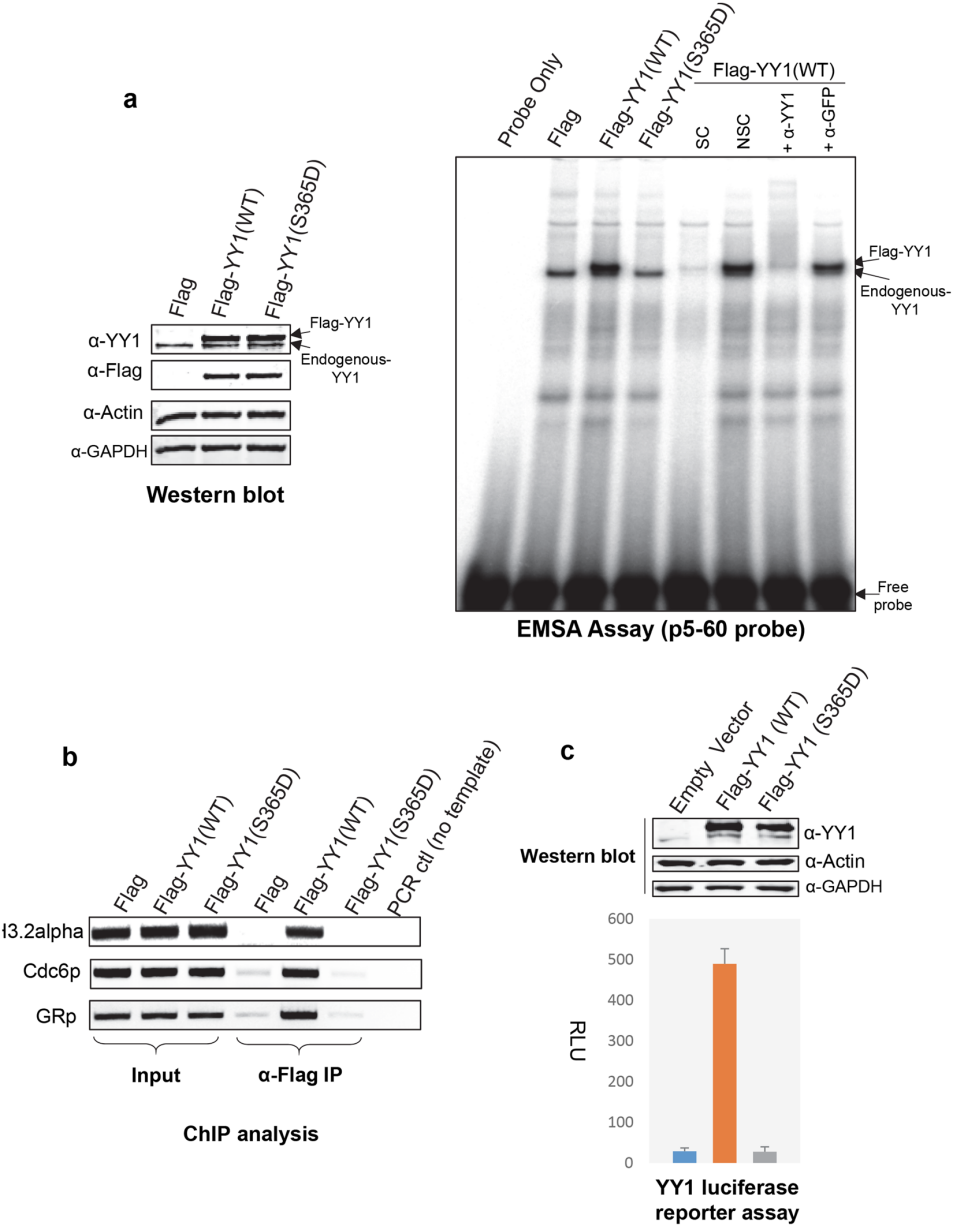
Serine 365 phosphorylation of YY1 abolishes its DNA binding and transcriptional activity in mammalian cells. **(a)** Flag or Flag-YY1 WT or S365D mutant were exogenously expressed in HeLa cells. Whole cell extracts were prepared and analysed by western blotting with α-YY1 and α-Flag antibodies to show the equal expression of the Flag-YY1 protein. Equal total protein loading was confirmed by probing with α-actin and α-GAPDH antibodies (left panel). The DNA binding activity of the expressed Flag-YY1 WT or S365D mutant were then tested in an EMSA assay with radioactively-labeled P5-60 probe (right panel). SC: specific-competition with unlabeled oligonucleotide; NSC: non-specific competition with unlabeled mutated oligonucleotide. **(b)** ChIP analysis of the *in vivo* binding of Flag-YY1 WT or S365D mutant in HeLa cells. Exogenously expressed Flag-YY1 was immunoprecipitated with α-Flag antibody. Three known *in vivo* YY1 binding sites were amplified by PCR**:** the alpha element in the histone H3.2 coding region, the Cdc6 promoter, and the glucocorticoid receptor promoter (GRp). The PCR reactions were analyzed on an agarose gel and DNA was stained with ethidium bromide **(c)** pCS2(+)Flag or pCS2(+)Flag-YY1 WT or S365D mammalian expression plasmids were co-transfected into HEK293 cells with a YY1 luciferase reporter plasmid P5 + 1-tk-luc^33^. 24 hours post-transfection, cells were collected analysed for luciferase signal (normalised to total protein levels). The graph shows the average signal from 4 independent transfections.

Next we wanted to confirm that this modification can abolish YY1’s DNA binding activity in human cells. We sub-cloned the YY1 S365D mutant into a mammalian expression plasmid, C-terminal to a Flag tag. As shown in the western blot analysis in Fig. 6a (left panel), the Flag-YY1-S365D mutant expressed equally to the WT Flag-YY1 in HeLa cells. However, the YY1 mutant was not able to bind DNA when tested in EMSA assays using HeLa whole cell lysates (Fig. 6a, right panel and Supplementary Figure 2). Note, that the exogenously expressed Flag-YY1 results in slightly higher shift that can be distinguished from the endogenous YY1 shift. Then we tested whether this phosphorylation could abolish the interaction of YY1 with genomic binding sites *in vivo*. For this, we performed chromatin immunoprecipitation (ChIP) assays on HeLa cells exogenously expressing Flag-YY1 WT or the S365D mutant. We examined the binding of Flag-YY1 to three established *in vivo* YY1 genomic binding sites within the coding region of histone H3.2^45^, the *Cdc6* promoter^46^, and the glucocorticoid receptor (*GR*) promoter^47^. While Flag-YY1 WT was efficiently associated with these genomic locations, the S365D mutant did not show any direct association (Fig. 6b).

Finally, in conformity with the complete loss of DNA binding activity, the YY1 S365D mutant was not able to activate gene expression from a luciferase reporter plasmid containing a YY1 binding site^33^ (Fig. 6c). Taken together, the results of these experiments, clearly show that Aurora A phosphorylation inactivates the DNA binding activity, and consequently the transcriptional activity, of YY1.

### Serine 365 phosphorylation overlaps, but is not needed for, the linker phosphorylation of YY1

**Figure 7:**
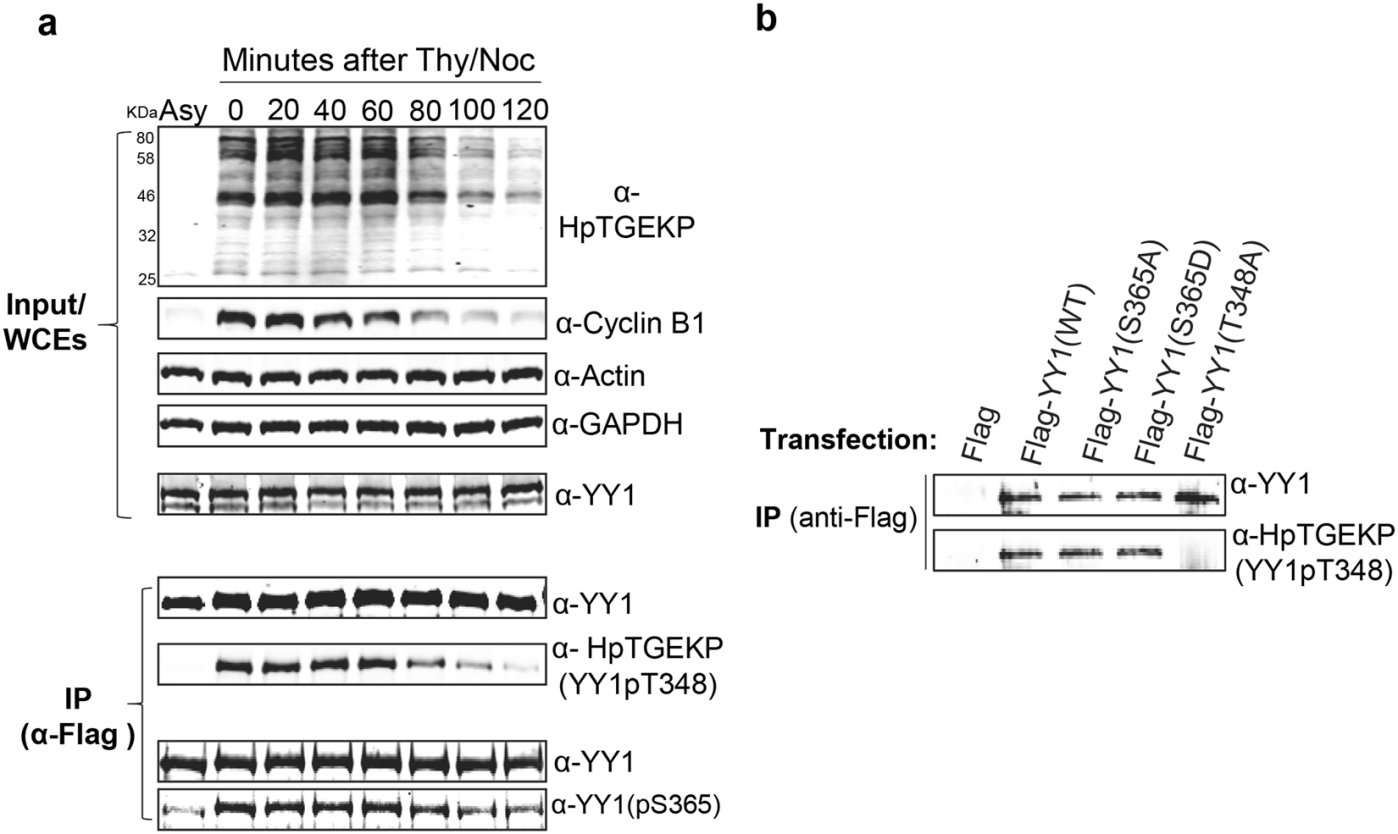
Overlap of multiple phosphorylation sites on YY1 DNA-binding domain during mitosis. **(a)** HeLa-Flag-YY1 cells were synchronised by a thymidine/nocodazole protocol. After removal of the nocodazole containing media and washing the cells, they were seeded in different plates, in fresh media to release from the block and progress through mitosis. Cells were collected at 20 minutes time-points after the release. Whole cells extracts were prepared and analysed by western blotting as indicated in the top panel. Flag-YY1 was immunoprecipitated and split into two different fractions, analysed separately using western blotting as indicated. **(b)** Flag or Flag-YY1 WT, S365A, S365D, or T348A were exogenously expressed in HeLa cells. 24 hours post-transfection, nocodazole (100ng/ml) was added for 18 hours. Whole cell extracts were then prepared and subjected to immunoprecipitation with anti-flag antibody (resin M2). The precipitated immune-complexes were analysed by western blotting with α-YY1 antibody to show equal YY1 levels, and α-HpTGEKP antibody, which recognised the YY1 phosphorylation on the second linker motif. Note the loss of the α-HpTGEKP signal on Flag-YY1(T348A).

YY1 belongs to the family of C2H2 zinc finger proteins, which represent the largest family in the human proteome. This family of proteins contain highly-conserved small linker sequences that connect their consecutive zinc finger domains^48, 49^. The linkers play an important role in the DNA binding efficiency of multi-zinc finger proteins. Using a phospho-specific antibody against the consensus linker sequence (anti-HpTGEKP), we have previously shown that these linker motifs are phosphorylated in mitosis, very specifically from mid-prophase until telophase^50^. We have identified TOPK (aka PBK) as the kinase responsible for this phosphorylation^51^. As a C2H2 zinc finger protein, we have shown that YY1 also carries this mitotic phosphorylation^50^. On the other hand, serine 365 phosphorylation is an inactivation mechanism specific to YY1. Here, we wanted to examine the temporal overlap of these phosphorylation events and whether serine 365 phosphorylation can affect the linker phosphorylation of YY1. HeLa-Flag-YY1 cells were synchronised by thymidine/nocodazole block, and then released to progress through mitosis. Cells were collected at 20 minute time-points after the release; whole cell extracts were prepared and analysed using western blotting. As shown in Fig. 7a (top panels) the linker phosphorylation (HpTGEKP/Threonine 348 of YY1) is detected on a large number of proteins and is very abundant in nocodazole-arrested cells. This phosphorylation starts decreasing at the 80 minute time point (start of telophase), directly following the decrease in Cyclin B levels which occurs during the metaphase-anaphase transition^1^ (starts at 40–60 minute time-points). The timing of the loss of the general HpTGEKP signal in whole cell extracts is similar to that observed on Flag-YY1 immunoprecipitated from these extracts. Examining the S365 phosphorylation on Flag-YY1 shows a significant temporal overlap, and it also decreases towards the later time points. However, the S365 phosphorylation appears to persist slightly longer towards the exit of mitosis (Fig. 7a, lower panels). Examining the HpTGEKP phosphorylation on YY1 WT versus non-phosphorylatable S365A mutant showed no detectable difference (Fig. 7b). This indicates that the YY1 S365 phosphorylation does not appear to have an effect on the YY1 linker phosphorylation. It is not clear at this moment whether these two phosphorylation events occur on the same YY1 molecule or different YY1 pools. The persistence of a small amount of S365 phosphorylation towards the end of mitosis indicates a potentially distinctive function in the inactivation of a specific pool of YY1 in telophase. It would be interesting in future studies to investigate whether this modification can modulate YY1’s role in the M/G1 reactivation of gene expression.

## Discussion

Even today, we know very little about the mitotic regulation of transcription factors, particularly in comparison to the finely dissected mechanisms of chromosome segregation. Transcription factors were initially thought to be excluded from condensed DNA during mitosis^7^. However, later studies have shown that mitotic chromosomes could actually be accessible to transcription factors^52^. Moreover, a few reports over the past two decades have shown that some transcription factors (like Oct-1, GHF1, Sp1, and Ikaros) transiently lose their DNA binding activity during mitosis^8–11, 50, 53^. Although phosphorylation of the DNA-binding domain has been shown to be the primary underlying mechanism of this inactivation, the responsible kinase(s) remained unknown. Identification of the signaling pathways and the kinases responsible for the mitosis-specific inactivation of transcription factors is critical to understand how this regulation is coordinated and synchronised with the other mitotic steps.

We have recently shown that the serine/threonine kinase TOPK is responsible for the mitotic inactivation of C2H2 zinc-finger transcription factors, through phosphorylation of their linker motifs^51^. Linker motifs are critical for the DNA binding activity of this family of transcription factors and their mitotic phosphorylation is tightly synchronised with chromosome condensation^50^. The identification of TOPK, which is a Cdk1 substrate and only active during mitosis, explains the mitotic exclusivity of this inactivation mechanism and its synchronisation with chromosome condensation.

In this report, we uncover a novel phosphorylation mechanism implicating Aurora A in the mitotic regulation of the transcription factor YY1. We provide solid evidence that YY1 is phosphorylated by Aurora A during mitosis and that this phosphorylation inactivates its DNA binding activity. We used purified proteins in *in vitro* kinase assays to show that YY1 is a direct substrate for Aurora A. We mapped the Aurora A phosphorylation site on YY1 to serine residue 365 in the third zinc finger of the DNA-binding domain. Investigation of this phosphorylation site in human cells, using a novel phospho-specific antibody, showed that it is highly specific to mitosis, correlating with the high peak of Aurora A levels and activity. Inhibition of Aurora A activity in mitotic cells by a small-molecule inhibitor or siRNA knockdown resulted in significant reduction of YY1 serine 365 phosphorylation. This supports the *in vitro* data that serine 365 residue of YY1 is an Aurora A phosphorylation site. Moreover, using electrophoretic mobility shift assays, we showed that YY1 phosphorylation by Aurora A or a phosphomimetic mutation at serine 365 diminishes its DNA binding activity *in vitro*. Chromatin immunoprecipitation assays showed that a YY1 S365D mutant does not associate with YY1 binding sites in cellular chromatin. In conformity with this loss of DNA binding activity, we showed that phosphomimetic mutation of YY1 at serine 365 abolishes its trans-activating function in a luciferase reporter assay.

Aurora kinase A is one of the key mitotic kinases. The expression and activity of Aurora A is tightly connected to other critical mitotic kinases like Cdk1 and Plk1 through several layers of feedback loops^54, 55^. Aurora A has pleiotropic effects on cell division. It is involved in the control of mitotic entry through centrosome maturation and spindle assembly. Moreover, Aurora A activity is also needed for cytokinesis and successful exit from mitosis^17, 18, 56^. Recently, Aurora A has been also implicated in the mitosis-specific regulation of transcription factors through phosphorylation of RUNX3^14^. Our findings here that Aurora A also phosphorylates YY1 and inactivates its DNA binding activity corroborate this role.

Interestingly, in addition to the loss of association with chromatin, the Aurora A phosphorylation of RUNX3 facilitates its redistribution to other mitotic structures, like the centrosome and midbody^14^. These findings revealed a new function for RUNX3 in the regulation of mitosis, triggered by Aurora A phosphorylation. YY1 has also been shown to be needed for proper completion of mitosis. Knockdown of YY1 leads to defects in cytokinesis and the accumulation of multinucleated cells^27^. However, this phenotype has been attributed to YY1’s role as a transcription factor during G2/M. It would be very interesting if the Aurora A phosphorylation of YY1 could also induce mitosis-specific protein interactions and an active role in the mitotic process. In addition to their function in DNA binding, the zinc finger domains are also important for protein-protein interactions. Since Aurora A phosphorylates the third zinc finger of YY1, it is likely that it would impact its protein interactions. The possibility of a direct, non-transcriptional, involvement of YY1 in the process mitosis is currently under investigation in our laboratory.

## Materials and Methods

### Cell Culture

HeLa S3 and HEK293 cells were cultured at 37 °C in 5% CO_2_ in Dulbecco’s Modified Eagle Medium (DMEM) (Cellgro, Herndon, VA) supplemented with 10% Fetal Bovine Serum (FBS) (Mediatech, Herndon, VA), and 1% Penicillin-Streptomycin (Mediatech). HeLa-Flag-YY1 cells are stably transfected with pCS2(+)-Flag-YY1, the generation of this cell line has been previously described^28^. Cells were arrested at pro-metaphase of mitosis by adding nocodazole (100 ng/ml) or Taxol (100 nM) (Sigma) for 16–18 hours; cells were then collected by shake-off. For thymidine-nocodazole synchronisation, cells were blocked in thymidine (Sigma) at 2.5 mM for 18 hours, released for 3 hours, and then nocodazole was added for 12 hours. Cells were then collected, washed, and re-plated into fresh media to progress through mitosis. For the inhibition of Aurora A activity, VX-680 (LC Laboratories, Woburn, MZ) was added, for 15 minutes, to cells blocked in mitosis by nocodazole. For Aurora A knockdown, cells were transfected with control scrambled siRNA or Aurora A siRNA (50 nM) using DharmaFECT reagent (Dharmacon, Chicago, IL). After 36 hours of knockdown, the cells were blocked in mitosis with nocodazole for 18 hours. For transfection of mammalian expression plasmids, the Polyjet transfection reagent (SignaGen, Rockville, MD) was used according to the manufacturer’s instructions.

### Whole cell extract (WCE) preparation and immunoprecipitation

Cells were washed with ice-cold PBS, and lysed in freshly prepared lysis buffer (50 mM Tris pH 8.0, 100 mM NaCl, 0.5% Triton-X 100, 1 mM EDTA, 10 mM NaF, 10 mM β-glycerophosphate, 10 mM sodium orthovanadate, and a cocktail of protease inhibitors), for 15 minutes on ice. The crude lysates were then cleared by centrifugation for 15 minutes at 4 °C. The cleared lysates were quantified using Bradford assay (Bio-Rad). To immunoprecipitate Flag-YY1, WCEs were incubated for 2 hours at 4 °C with a mouse monoclonal anti-Flag antibody coupled to agarose beads (Resin M2, Sigma). Resin M2-Flag-YY1 complex was then washed three times with lysis buffer, then processed for Western blotting.

### Western blotting

Proteins were separated on a SDS-PAGE gel and transferred to a nitrocellulose membrane. After transfer, the membrane was blocked in TBSTM (Tris-buffered saline, 0.5% Tween20, 5% Milk) for 30 minutes. Probing with the indicated primary antibodies in blocking solution was for 2 hours at room temperature (RT) or overnight at 4 °C. Donkey anti-mouse (IRDye 680LT) or anti-rabbit (IRDye 800CW) secondary antibodies (LiCOR) were added for 1 hour at RT. Blots were imaged using the LiCOR Odyssey system. Primary antibodies used for western blotting were anti-YY1 (H-10), anti-Cyclin B1, and anti-GAPDH, anti-GST, anti-Actin (Santa Cruz Biotechnology, Santa Cruz, CA), anti-Aurora A (D3E4Q) (Cell Signaling Technology, Danvers, MA), and anti-Flag (Sigma). Anti-HpTGEKP antibody was previously described^50^. The rabbit polyclonal anti-YY1pS365 was generated by New England Peptide Inc. (Gardner, MA) using a synthesised phospho-peptide corresponding to YY1 amino acids 360–369 (Ac-CGKRF(p)SLDF-amide).

### Plasmids

The construction of pCS2(+)-Flag-YY1 and pGEX-2T-YY1 (full length and deletion mutants) mammalian and bacterial expression plasmids was previously described^28, 31^. The YY1 deletion mutants that were subcloned into pGEX-2T vector were originally a generous gift from Dr. Bernhard Lüscher^33^. Generation of YY1 point mutations at position 365 from serine to alanine or aspartic acid was performed using the pET-20b( + )-YY1 plasmid^31^. The YY1 ORF was then subcloned into pGEX-2Tand pCS2( + )-Flag vectors as described previously^28, 31^. Mutagenesis was performed using the QuickChange Lightning Site-Directed Mutagenesis Kit (Agilent Technologies, La Jolla, CA) according to manufacturer’s instructions. The primers used for the mutagenesis of serine 365 were designed using the QuikChange Primer Design Program on the Stratagene Web site. All mutations were confirmed by sequencing.

### Bacterial expression and purification

Bacterial expression of GST-YY1 and the expression and purification of non-tagged YY1 were performed as previously described^31^.

### *In vitro* kinase assays

Kinase reactions were performed in kinase buffer (50 mM Tris pH 7.4, 10 mM MgCl_2_, 50 µM ATP, 0.25 µM^32^P-γ-ATP, 5 mM beta-glycerophosphate, 10 mM NaF, 1 mM DTT) for 30 minutes at 30 °C, with shaking. Purified active Aurora A kinase was purchased from SignalChem (British Columbia, Canada). Reactions were then stopped by the addition of SDS-PAGE buffer and loaded for separation on a 10% SDS-PAGE gel. After staining with Coomassie Brilliant Blue R-250, to visualise the protein bands, gels were dried and exposed overnight to a phosphorimager screen at room temperature. The screen was then scanned on a Typhoon 9410 imager (GE Healthcare, Waukesha, WI) for analysis. For the cold kinase assays, no radioactive ATP was added, and the cold ATP concentration was raised to 2 mM. After separation on the SDS-PAGE gel, proteins were transferred to a nitrocellulose membrane and probed with the indicated antibodies.

### Electrophoretic Mobility Shift Assay (EMSA)

Double-stranded DNA oligonucleotides (Integrated DNA Technologies) were end-labeled using T4-polynucleotide kinase (New England Biolabs) and [γ-^32^P]ATP (Perkin Elmer-Cetus, Boston, MA). EMSA conditions were as previously described^45^, except that the native gels were 8% polyacrylamide and the dry gels were exposed to a phosphorimager screen and scanned using a Typhoon 9410 Imager (GE Healthcare). The oligonucleotides used as probes contain variant sequences of the YY1 binding sites in the Histone genes coding regions (H3.2α)^45^ and the promoter of the adeno-associated virus AAV (P5 + 1 and P5-60)^44^. The specificity of the YY1 shifts was verified by incubating the reactions with unlabeled oligonucleotides. For non-specific competition, the reactions were incubated with a mutated H3.2alpha oligonucleotide (lacking the CAT core YY1 binding motif). For the EMSA assays testing the HeLa whole cell extracts, we used additional specificity controls by incubating the reactions with anti-YY1 (H10) mouse monoclonal antibody. Anti-GFP mouse monoclonal antibody was used as a negative control.

### Chromatin Immunoprecipitation (ChIP)

ChIP was adapted from the protocol described previously by Boyd, *et al*.^57^. HeLa cells transfected with pCS2(+)-Flag, pCS2(+)-Flag-YY1 WT or S365D plasmids were cross linked in 1% formaldehyde and the reaction was quenched with glycine. After two washes with cold PBS, cells were scraped in 0.5 ml swelling buffer and incubated on ice 10 min. Pelleted cells were resuspended in lysis buffer and incubated on ice 10 min. Chromatin DNA was sonicated and centrifuged for 10 minutes at 13,000 rpm at 4 °C. Flag-YY1 was immunoprecipitated from the cleared lysates for 2 hours with a mouse monoclonal anti-Flag antibody coupled to agarose beads (Resin M2, Sigma). After washing and elution, the protein-DNA complex was reversed by heating at 65 °C for 4 hours. Eluate was adjusted to 40 mM Tris pH 6.8, 10 mM EDTA, then incubated with RNase A, and followed by proteinase K. DNA was recovered by phenol:chloroform extraction. PCR was performed with MasterMix from 5 Prime with the following primer sets: H3.2 coding region 5′-ATTCCAGCGTCTGGTACGTGAGAT-3′ (forward) and 5′-ACTTATGCCCTCTCTCCGCGAAT-3′ (reverse), cdc6 promoter 5′-AAA GGC TCT GTG ACT ACA GCC AAT-3′ (forward) and 5′-GTG CAG GAT CCT TCT CAC GTC TCT CAC-3′ (reverse), and GR promoter 5′-CTT TTC CGA GGT GGC GAG TAT C-3′ (forward) and 5′-CCC CCT GCT CTG ACA TCT T-3′ (reverse).

### Luciferase Assay

The YY1 luciferase reporter plasmid (P5 + 1-tk-luc)^33^ is a kind gift from Dr. Bernhard Lüscher. P5 + 1-tk-luc was co-transfected with pCS(2) + Flag, pCS(2) + Flag-YY1 WT or S365D mutant into HEK293 cells for 24 hours. Cells were then processed using the Promega Luciferase assay kit. Means and standard deviations from a representative experiment of four replicates are shown. Equivalent expression of Flag-YY1 WT or S365D was confirmed by western blotting with anti-YY1 antibody.

### Flow Cytometric Analysis

Cells were washed two times with PBS, and then fixed in 70% ethanol on ice for at least 2 hours. After washing off the ethanol, cells were resuspended in propidium iodide (PI) solution (50 µg/ml PI, 200 µg/ml RNase A, 0.1% Triton-X 100 in PBS) and incubated for 30 min at 30 °C. The cell suspension was then passed through a 50 µm nylon mesh to remove clumps. Cells were then analysed based on DNA content on a fluorescence-activated cell analyser (Canto; Becton Dickinson, San Jose, CA), 10,000 cells were counted for each sample. Images were generated using BD Diva software.

### Availability of data and materials

No datasets were generated or analysed during the current study.

## Electronic supplementary material

Supplementary Data
