# Supplementary material for: Aurora A Phosphorylation of YY1 during Mitosis Inactivates its DNA Binding Activity
Source: Sci Rep. 2017 Aug 30;7:10084. doi: 10.1038/s41598-017-10935-5 (PMC5577188; doi:10.1038/s41598-017-10935-5)
Supplement: Supplementary file 1 — Supplementary Data [file 41598_2017_10935_MOESM1_ESM.pdf]

Aurora A Phosphorylation of YY1 during Mitosis Inactivates its DNA-Binding Activity

Karen E. Alexander[1] and Raed Rizkallah[1*]
[1] Department of Biomedical Sciences, Florida State University, Tallahassee, Florida, United States of America.

[*] Correspondence:
Tel: (850) 645-0779
Fax: (850) 645-7153
Email: raed.rizkallah@med.fsu.edu

Supplementary Figures

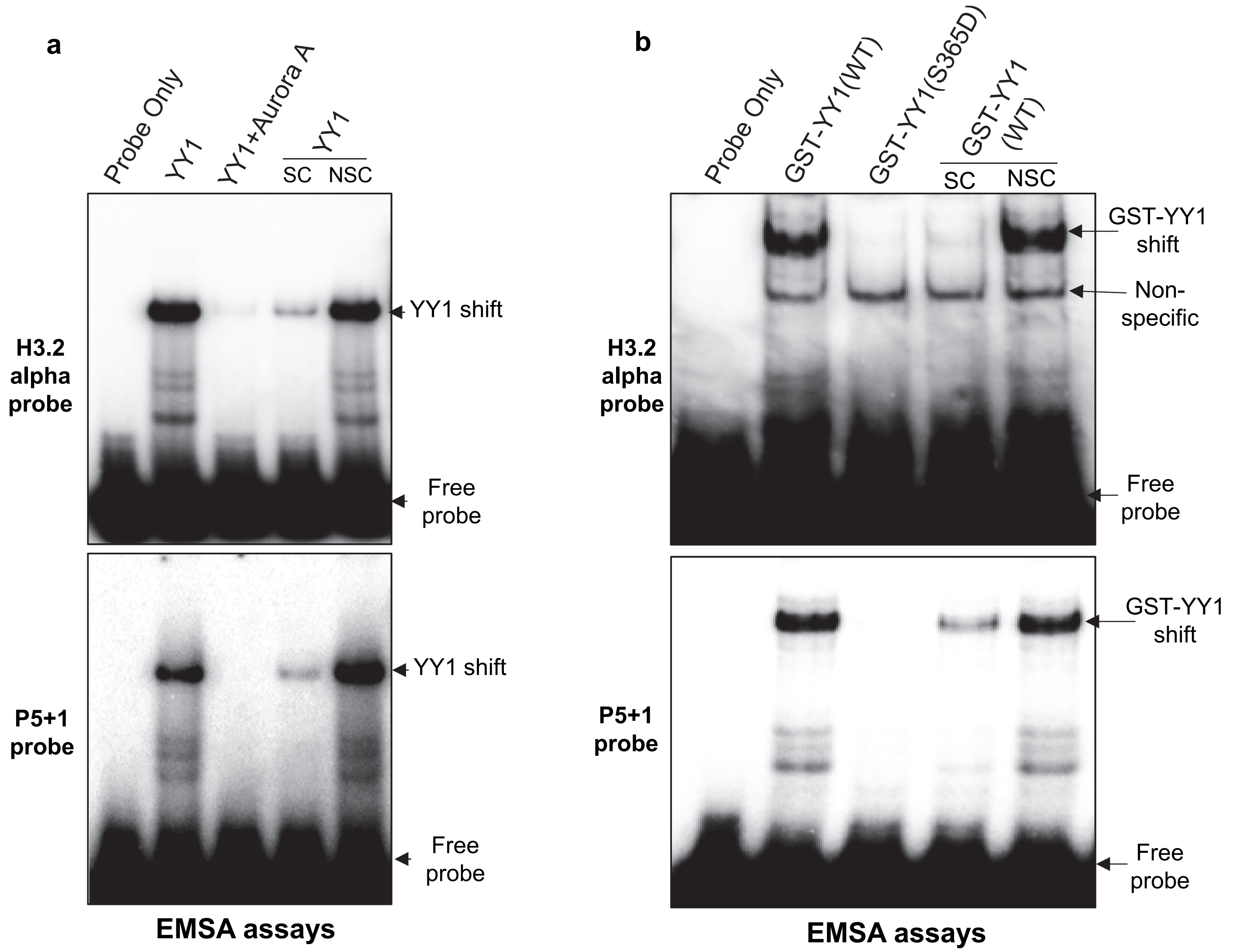

Supplementary figure 1. YY1 phosphorylation at serine 365 by Aurora A abolishes its DNA-binding activity. (a) Bacterially-expressed and purified YY1 was used as a substrate in *in vitro* kinase reactions, with or without active Aurora A. A fraction of the reactions were tested by western blotting for equal YY1 levels and YY1 pS365 phosphorylation (Figure 5b, upper panel). The remaining fractions of the kinase reactions were incubated with radioactively labeled double–stranded DNA oligonucleotides containing different variants of the YY1 consensus binding sites, as labelled. **(b)** GST-YY1 WT or S365D were expressed in bacterial BL21 cells. Bacterial lysates were tested by western blotting for equal YY1 levels (Figure 5c, upper panel). Corresponding amounts of the bacterial lysates were then tested in EMSA assays for YY1 binding activity as in (a). SC: specific-competition with unlabeled oligonucleotide; NSC: non-specific competition with unlabeled mutated oligonucleotide.

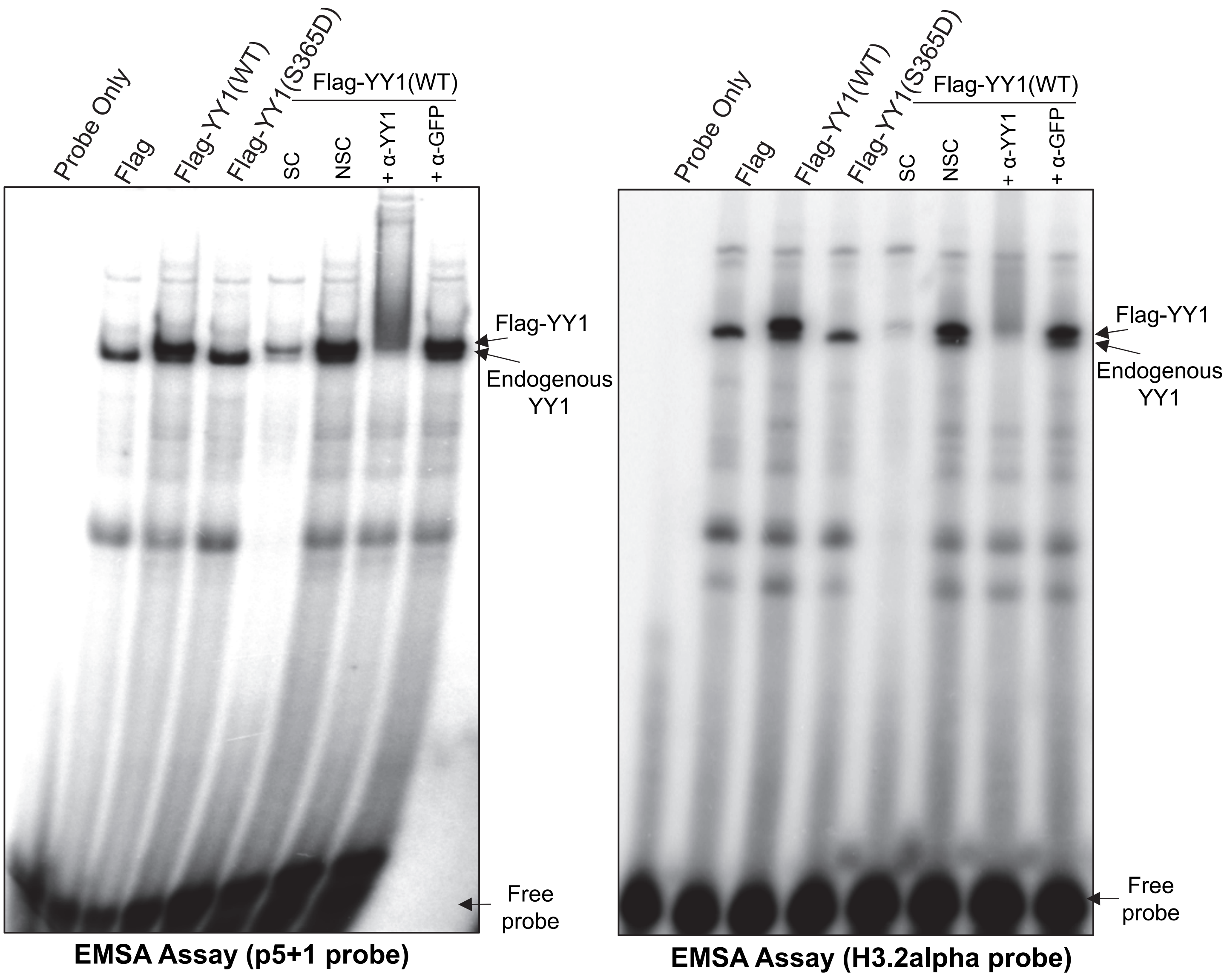

Supplementary figure 2. Serine 365 phosphomimetic mutation abolishes the DNA-binding activity of YY1 in mammalian cells. Flag or Flag-YY1 WT or S365D mutant were exogenously expressed in HeLa cells. Equal expression of Flag-YY1 WT and mutant were verified with western blot analysis (Figure 6a, left panel). The DNA-binding activity of the exogenously expressed Flag-YY1 WT or S365D mutant were then tested in EMSA assays with the radioactively-labeled probes p5+1 and H3.2alpha, as labeled. SC: specific-competition with unlabeled oligonucleotide; NSC: non-specific competition with unlabeled mutated oligonucleotide.
